# Paradoxical spinopelvic motion: does global balance influence spinopelvic motion in total hip arthroplasty?

**DOI:** 10.1186/s12891-021-04865-7

**Published:** 2021-11-23

**Authors:** Yu-Hsien Lin, Yu-Tsung Lin, Kun-Hui Chen, Chien-Chou Pan, Cheng-Min Shih, Cheng-Hung Lee

**Affiliations:** 1 Department of Orthopedics, Taichung Veterans General Hospital, Taichung, Taiwan; 2 College of Medicine, National Chung Hsing University, Taichung, Taiwan; 3 Department of Computer Science and Information Engineering, Providence University, Taichung, Taiwan; 4 Department of Rehabilitation Science, Jenteh Junior College of Medicine, Nursing, and Management, Miaoli County, Taiwan; 5 Department of Physical Therapy, Hung Kuang University, Taichung, Taiwan; 6 Department of Food Science and Technology, Hung Kuang University, Taichung, Taiwan

**Keywords:** Paradoxical spinopelvic motion, Spinopelvic stiffness, Global spinal alignment, Sagittal alignment, Dislocation, Total hip arthroplasty

## Abstract

**Background:**

Recent research has proposed a classification of spinopelvic stiffness according to pelvic spatial orientation for risk stratification in patients who undergo total hip arthroplasty (THA). However, the influence of global alignment was not investigated, and this study evaluated the effect of global balance (sagittal vertical axis [SVA]) on spinopelvic motion.

**Methods:**

We conducted a retrospective review of consecutive primary THA patients. We measured SVA, spinopelvic parameters (pelvic tilt [PT], pelvic incidence, and sacral slope), thoracic kyphosis (TK), lumbar lordosis (LL), proximal femur angle (PFA), and cup version using functional radiographs of patients in the standing and upright sitting positions. Linear regression was performed to identify parameters related to global trunk alignment change (∆SVA). Spinopelvic stiffness was defined as PT position change < 10°, and a subset of patients with PT change < 0° was categorized into a paradoxical spinopelvic motion group.

**Results:**

One hundred twenty-four patients were analyzed (mean age: 65 years, 61% female). In univariate regression analysis, ∆TK, ∆LL, and ∆PFA were correlated to ∆SVA. In multivariate regression analysis, ΔLL (*p* < 0.001) and ΔPFA (*p* < 0.001) were found to be correlated to ΔSVA (ΔSVA = − 11.97 + 0.05ΔTK – 0.23ΔLL – 0.17ΔPFA; adjusted R^2^ = 0.558). Spinopelvic stiffness was observed in 40 patients (32%), including five (4%) with paradoxical motion (∆PT = − 3° ± 1°, *p* < 0.001) with characteristics of balanced standing global trunk alignment (standing SVA = − 1.0 ± 5.1 cm), similar stiffness of the lumbosacral spine (∆LL = − 7° ± 5°), higher hip motion (∆PFA = − 78° ± 6°, *p* = 0.017), and higher anterior trunk shift (∆SVA = 6.2 ± 2.0 cm, *p* = 0.003) from standing to sitting as compared to the stiffness group. Two of these five patients experienced dislocation events after THA.

**Conclusions:**

The lumbosacral and hip motions were the major contributors to global alignment postural change. Paradoxical motion is a rare but dangerous clinical condition in THA that might be related to a disproportionally large trunk shift in the stiff lumbosacral spine causing excessive hip motion. In paradoxical motion, diminishing functional acetabular clearance during position change might pose the prosthesis at higher risk of impingement and instability than spinopelvic stiffness.

## Background

**Fig. 1:**
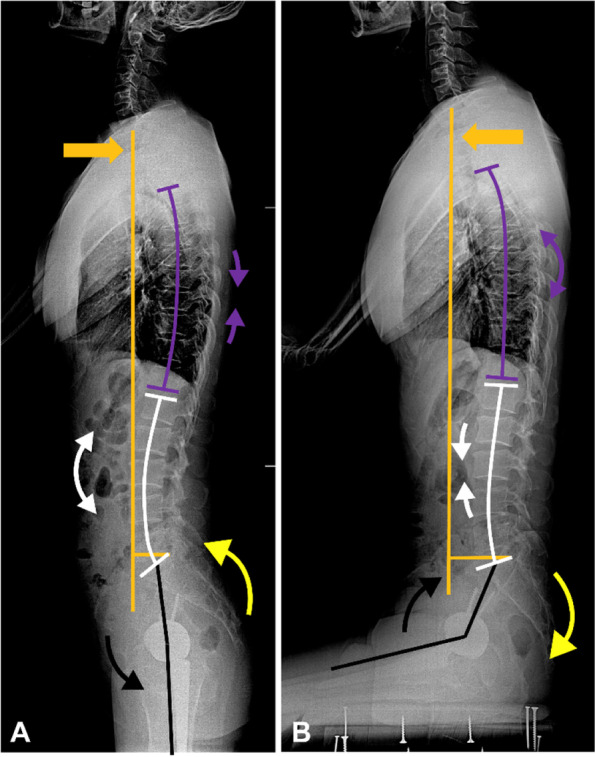
The normal spinopelvic motion from standing (**A**) to sitting (**B**) position includes thoracic flexion (purple arrow), lumbar flexion (white arrows) and hip flexion (black arrow), which allows trunk anterior shift (orange arrow) and pelvic retroversion (yellow arrow). The pelvic retroversion provides acetabular anterior clearance during hip flexion

Recent studies have revealed that the Lewinnek safe zone [1] cannot effectively predict dislocation after total hip arthroplasty (THA) [2, 3]. Abnormal spinopelvic motion [4–8] is an essential cause of failure of Lewinnek safe zone. In normal spinopelvic motion (Fig. 1), switching from standing to a sitting position will result in the flattening of the lumbar-sacral complex [4, 6–8]. This motion includes flexion (kyphosis) of the lumbosacral spine (about 25°-30°) [9], which leads to approximately 20° pelvis retroversion and increases the acetabulum’s anteversion by approximately 14° [10]. Under the normal spinopelvic motion, the sitting position can be reached with only 50° hip joint flexion. Finally, this will also affect global trunk balance, resulting in an anterior shift of the body’s center of gravity [11–13] to reach a stable upright sitting position.

Spinopelvic stiffness can cause THA impingement and instability [4–8]. Reduced lumbosacral mobility due to a degenerative disease or iatrogenic fusion of the lumbar-sacral motion segments causes compensatory recruitment of the hip motion for the patient’s posture changes. However, the reduced lumbosacral motion will prevent the pelvis from providing the corresponding anterior or posterior clearance for the femur. This may result in THA impingement and instability. According to the classification by Stefl et al. [4] and related studies [4, 6, 8], spinopelvic stiffness can be divided into three clinical conditions based on the spatial orientation where the pelvis was fixed, namely, stuck sitting, stuck standing, and neutral stiffness conditions. However, the interrelationship between global alignment and spinopelvic motion was not yet investigated.

### Purpose

The main purposes of this study were to analyze the interrelationship between global trunk alignment and spinopelvic motions in a group of patients who underwent THA and identify spinopelvic motions with high risk to provide clinical guidance for component implantation.

## Materials and methods

Our institutional review board approved the conduct of this study. This was a retrospective review of coronal and sagittal spinal-pelvic digital triple-films of 156 consecutive patients who underwent primary THA performed by five independent orthopedic surgeons at one institute from January 2017 to December 2018. Spinopelvic radiographs were taken in patients after THA, including films taken in standing and upright relaxed sitting positions, from the cranial base proximally to the proximal femur distally. While in the upright sitting position, patients were requested to sit on a square platform with their legs suspended, and patients were requested to abduct their thighs by approximately 45° for cup version measurement. Exclusion criteria were as follows: scoliosis with Cobb’s angle > 10° (which affects the cup version [12, 13]), spinal ankylosis (which causes spinopelvic stiffness [14]), congenital spinal deformity, patients with neuromuscular comorbidities, and poor image quality.

Basic data including age, sex, body mass index cup, head, and stem size were recorded. Each patient was followed up for at least a year after surgery to confirm if any dislocation events occurred.

### Radiographic measurement

**Fig. 2:**
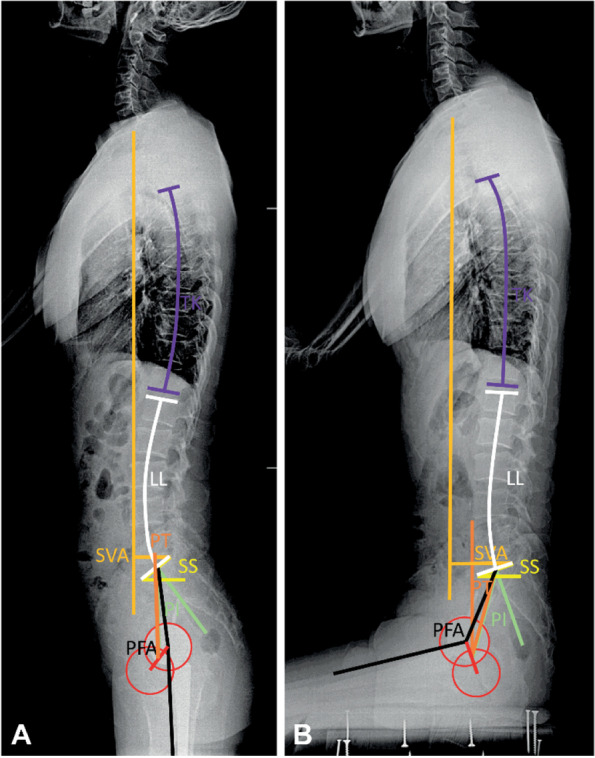
Sagittal parameters measured in this study, standing (**A**) and sitting (**B**). PT, pelvic tilt; SS, sacral slope; PI, pelvic incidence; TK, thoracic kyphosis; LL, lumbar lordosis angle; PI-LL, PI-LL miss-match; SVA, C7-sagittal vertical axis; PFA, pelvic-femoral angle

As shown in Fig. 2, sagittal spinal parameters measured on the standing and sitting sagittal views included three pelvic parameters, namely, PT, sacral slope (SS), and pelvic incidence (PI), as well as thoracic kyphosis (TK; T4-T12), lumbar lordosis angle (LL; L1-S1), PI-LL mismatch (PI-LL), C7-sagittal vertical axis (SVA) representing global alignment, and pelvic-femoral angle (PFA) [6, 7, 15, 16]. The Surgimap Ver. 2.3.1.1 software was used to measure all sagittal parameters.

Radiographic acetabular cup anteversion was calculated using the ellipse method [17], which has a high intra- and inter-observer reliability [3, 18]. Radiographic inclination was defined as the angle between the face of the cup and the sagittal plane [17].

### Statistical analysis

The normality of the radiographic data was confirmed using the Kolmogorov–Smirnov test. Parametric tests of the univariate linear regression analyses were performed using the dependent variable of the global alignment postural change (ΔSVA) with positional changes of sagittal parameters (ΔTK, ΔLL, ΔSS, ΔPT, ΔPFA) as independent variables and standing PI, owing to the constant nature of PI in each patient, using the forced data entry method (Δ: the sitting values minus the standing values). After identifying the sagittal parameters that correlated with global trunk alignment, a multivariate linear regression model was constructed.

To compare among groups, we used the Kruskal–Wallis test; a post hoc analysis was performed using the Dunn–Bonferroni test. The chi-squared test was used to compare categorical variables. Continuous variables were expressed as mean ± SD. A *P*-value of less than 0.05 indicated a statistically significant difference. All statistical calculations were performed using SPSS Statistics (version 22, IBM Corp., Armonk, New York).

## Results

From the total of 156 patients, we excluded 15 patients with scoliosis, two with spinal ankylosis, two with a neuromuscular disorder, and 13 due to poor image quality. There were 124 patients (48 males and 76 females) remaining, with an average age of 65 years. A total of 28 patients (23%) underwent spinal fusion before THA. The anterior-lateral approach was adopted for 21 patients (17%), while the posterior-lateral approach was used for 103 patients (83%).

Among the total 124 patients, 5 patients (4%) experienced dislocation within 1 year after THA, with the average time to dislocation of 7.2 weeks (2 weeks to 4 months). All five patients with dislocation were female and in the stiffness group (0° ≤ ∆PT < 10°) or the paradoxical motion group (∆PT < 0°) (Table 1). Among them, 4 underwent spinal fusion before THA, 2 had anterior, and 3 had posterior dislocation events.

**Table 1:** Basic demographics and comparison of radiographic parameters among three groups

	Normal Group ∆PT ≥ 10 (*n* = 84)		Stiffness group 0° ≤ ∆PT < 10° (*n* = 35)		Paradoxical group ∆PT < 0° (*n* = 5)		*p*-value
Sex							0.643
Female	50	(60%)	22	(63%)	4	(80%)	
Male	34	(40%)	13	(37%)	1	(20%)	
Age (year)	62	±15	69	±8	76	±15	0.076
Dislocation	0	(0%)	3	(9%)	2	(40%) ^a^	< 0.001**
Spinal fusion	11	(13%)	14	(40%) ^a^	3	(60%)	0.001**
Standing							
SVA (cm)	3.8	±4.5	4.3	±2.9	−1.0	±5.1	0.066
TK	25	±13	26	±10	25	±9	0.847
LL	47	±15	40	±15	50	±11	0.070
SS	35	±10	30	±10	33	±10	0.068
PT	13	±11	19	±9 ^a^	18	±8	< 0.001**
PI	48	±11	49	±10	51	±15	0.438
PI-LL	1	±15	9	±12 ^a^	1	±11	0.007**
PFA	184	±10	191	±11 ^a^	192	±9	0.004**
Cup anteversion	17	±9	19	±10	19	±4	0.734
Cup inclination	43	±7	45	±6	42	±4	0.476
Sitting							
SVA (cm)	6.0	±2.8	5.5	±2.6	5.2	±3.5	0.721
TK	25	±14	27	±10	27	±11	0.189
LL	22	±14	33	±13 ^a^	43	±6 ^a^	< 0.001**
SS	15	±11	24	±10 ^a^	35	±10 ^a^	< 0.001**
PT	33	±12	25	±9 ^a^	15	±8 ^a^	< 0.001**
PFA	133	±16	125	±12 ^a^	114	±9 ^a^	0.003**

*p < 0.05, **p < 0.01

^a^*p* < 0.05 compared to normal group

^b^*p* < 0.05 compared to stiffness group.

*SVA* C7-sagittal vertical axis, *TK* thoracic kyphosis, *LL* lumbar lordosis, *SS* sacral slope, *PT* pelvic tilt, *PI* pelvic incidence, *PI-LL* PI-LL mismatch, *PFA* proximal femur angle

In this study, we divided all patients as having normal spinopelvic motion (∆PT ≥ 10°, including normal variation of hypermobile spinopelvic motion (∆PT ≥ 30°)) and spinopelvic stiffness (∆PT < 10°) [19]. (Table 1) A small subset of patients with stiffness and a negative PT change (∆PT < 0°) was identified. Due to the opposite motion of the pelvis to the hip joint, such conditions were considered as paradoxical spinopelvic motions (Tables 1 and 2).

**Table 2:** Five patients with paradoxical spinopelvic motion

	Patient #1	Patient #2	Patient #3	Patient #4	Patient #5
Sex	Female	Male	Female	Female	Female
Age	70	101	72	61	76
Spinal fusion	No	No	L3–4 and L4–5	L4–5	T12-S2
Dislocation	Posterior dislocation	No	No	No	Posterior dislocation
Time to dislocation	1 month	No	No	No	4 months
Standing					
SVA (cm)	−1.9	−8.1	−2.8	5.4	2.2
TK	17	37	20	20	33
LL	52	61	56	47	33
SS	39	36	39	37	15
PT	12	16	23	28	11
PI	51	52	62	65	26
PI-LL	−1	−9	6	18	−7
PFA	182	191	196	206	187
Cup anteversion	21	22	13	16	21
Cup inclination	25	41	45	44	45
Sitting					
SVA (cm)	3.9	0.0	5.6	9.4	7.0
TK	14	41	17	29	35
LL	46	49	45	43	33
SS	44	38	40	39	18
PT	7	15	21	26	8
PFA	105	116	127	120	109
∆ (sitting-standing)					
SVA (cm)	5.8	8.1	8.4	4.0	4.9
TK	−2	4	−3	9	2
LL	−6	−13	−12	−4	0
SS	5	2	1	2	2
PT	−5	−2	− 2	−3	− 2
PFA	−77	− 79	−69	−86	−79

*SVA* C7-sagittal vertical axis, *TK* thoracic kyphosis, *LL* lumbar lordosis, *SS* sacral slope, *PT* pelvic tilt, *PI* pelvic incidence, *PI-LL* PI-LL mismatch, *PFA* proximal femur angle

### The interrelationship between global trunk alignment and spinopelvic motion

**Fig. 3:**
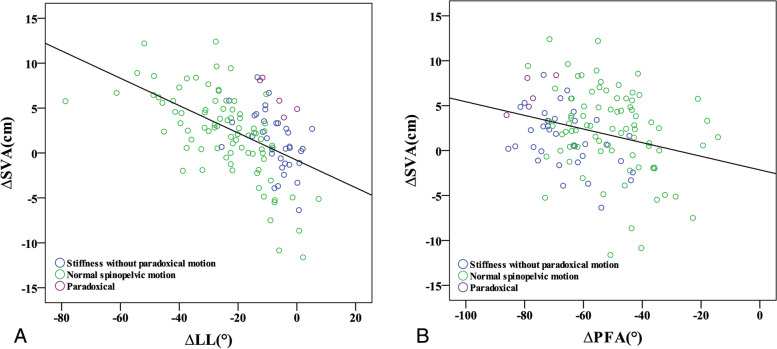
Scatter diagrams of ∆SVA to the parameters related to ∆SVA: ∆LL (3A), ∆PFA (3B). SVA, C7-sagittal vertical axis; LL, lumbar lordosis; PFA, proximal femur angle

Univariate linear regression analyses of the postural change of sagittal parameters (ΔTK, ΔLL, ΔSS, ΔPT, ΔPFA) and PI (constant in each patient) were performed to identify the parameters related to ΔSVA. ΔTK (*p* < 0.001), ΔLL (*p* < 0.001), and ΔPFA (*p* = 0.003) were correlated to ΔSVA (Table 3). The following model (adjusted R^2^ = 0.558) was constructed based on the results of multivariate linear regression analysis (Table 3 and Fig. 3):

**Table 3:** Liner regression analysis of ∆SVA and sagittal parameters

	Univariate			Multivariable		
	Unstandardized β coefficients	Standardized β coefficients	*p* value	Unstandardized β coefficients	Standardized β coefficients	*p* value
Constant				−11.97		< 0.001**
∆TK	−0.23	−0.36	< 0.001**	0.05	0.08	0.287
∆SS	−0.02	−0.06	0.495			
Standing PI	0.00	−0.01	0.925			
∆LL	−0.15	−0.53	< 0.001**	−0.23	−0.82	< 0.001**
∆PT	0.03	0.07	0.466			
∆PFA	−0.08	−0.27	0.003**	−0.17	−0.60	< 0.001**

(Multivariable regression model: ΔSVA = − 11.97 + 0.05ΔTK-0.23ΔLL – 0.17ΔPFA, Adjusted R^2^ = 0.558)

*p < 0.05, **p < 0.01

*SVA* C7-sagittal vertical axis, *TK* thoracic kyphosis, *LL* lumbar lordosis, *SS* sacral slope, *PT* pelvic tilt, *PFA* proximal femur angle, *PI* pelvic incidence

ΔSVA = − 11.97 + 0.05ΔTK - 0.23ΔLL - 0.17ΔPFA.

Notably, ΔTK was not a statistically significant factor (*p* = 0.287) in multivariate linear regression analysis. Therefore, our results indicate that lumbosacral (*p* < 0.001) and hip motions (*p* < 0.001) were the major contributors of global trunk shift during position change.

**Fig. 4:**
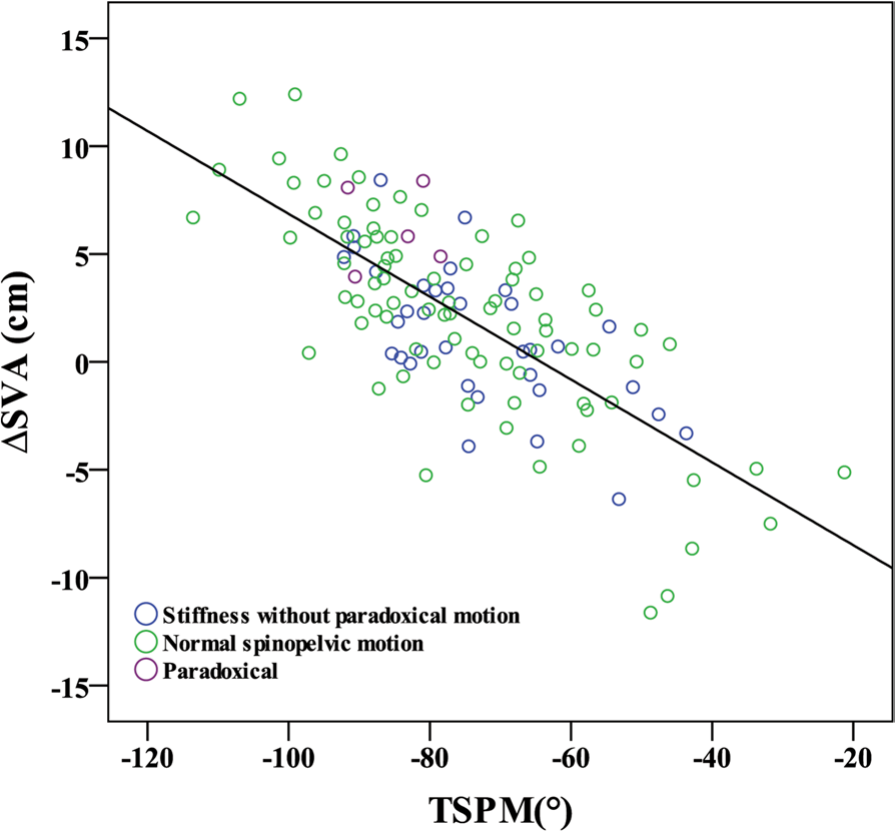
Scatter diagrams of ∆SVA to total spinopelvic motion (TSPM), which was the sum of lumbosacral(∆LL) and hip motion(∆PFA). LL, lumbar lordosis; PFA, proximal femur angle

**Fig. 5:**
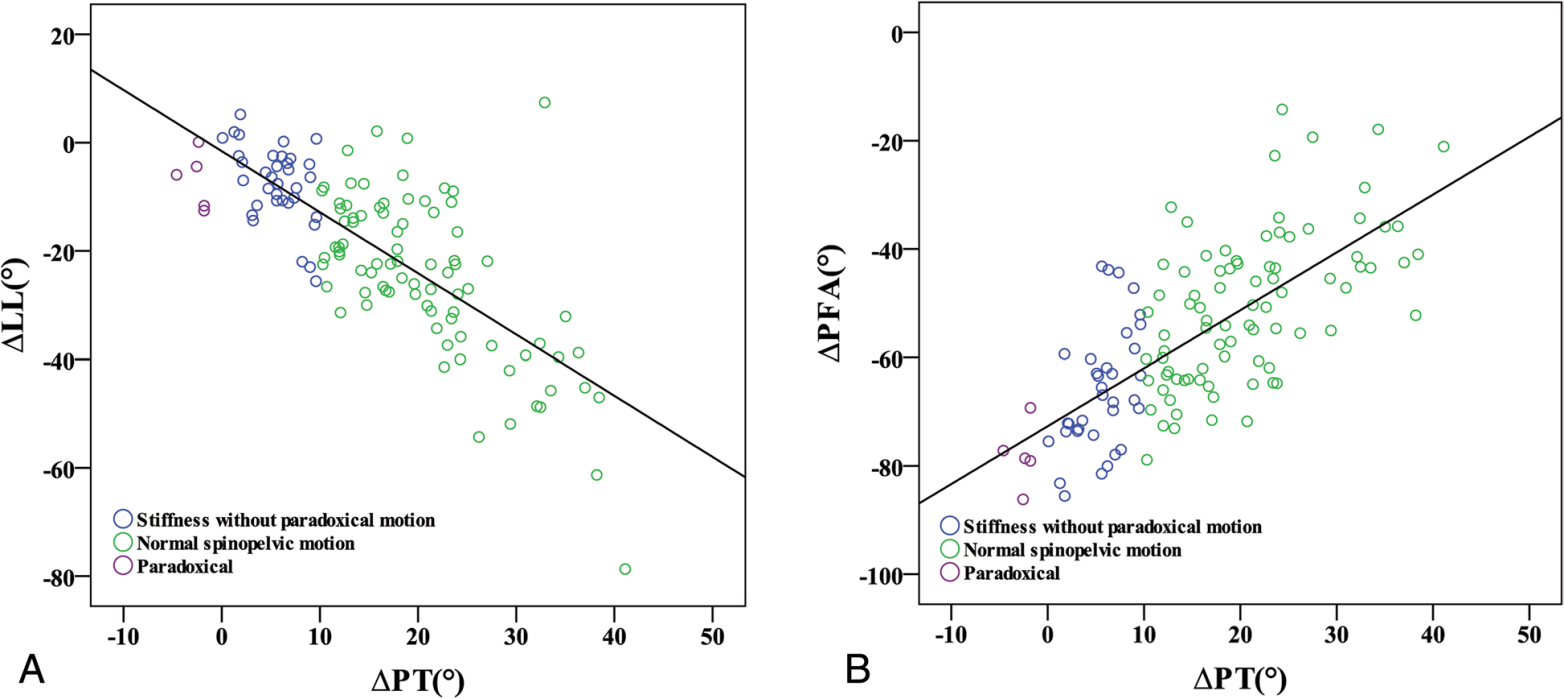
Scatter diagrams of ∆PT to ∆LL(A) and ∆PT to ∆PFA(B). LL, lumbar lordosis; PFA, proximal femur angle; PT, pelvic tilt

We named the sum of lumbosacral and hip motions as the “total spinopelvic motion (TSPM).” A Pearson correlation analysis of TSPM and ΔSVA (*p* < 0.001, R^2^ = 0.538) (Fig. 4) showed a moderate correlation, suggesting that a greater trunk shift during position change necessitates greater TSPM. The comparison of global alignment change, each part of the spinopelvic motion, and TSPM from standing to sitting among each group are shown in Table 4. Pearson correlation analyses of ΔLL to ΔPT (*p* < 0.001, R^2^ = 0.572), and ΔPFA to ΔPT (*p* < 0.001, R^2^ = 0.502) were also conducted to evaluate the effect of lumbar and hip motion to ΔPT (Fig. 5A and B). The result showed that ΔPFA smaller than − 72.7° and ΔLL higher than − 1.6° were both corresponding to ΔPT < 0°.

**Table 4:** Comparison of global alignment change and spinopelvic motion among each group from standing to sitting

	Normal Group ∆PT ≥ 10° (*n* = 84)		Stiffness Group 0° ≤ ∆PT < 10° (*n* = 35)		Paradoxical Group ∆PT < 0° (*n* = 5)		*P-*value
Difference (Sitting-standing)							
∆SVA (cm)	2.2	±4.7	1.3	±3.2	6.2	±2.0^b^	0.014*
TSPM	− 75	±18	−74	±12	−85	±6	0.216
∆LL	−25	±15	−7	±7^a^	−7	±5^a^	< 0.001**
∆PFA	− 51	±14	−66	±11^a^	−78	±6^ab^	< 0.001**
∆PT	21	±8	6	±3^a^	−3	±1^ab^	< 0.001**

*p < 0.05, **p < 0.01

^a^*P* < 0.05 compared to normal group

^b^*P* < 0.05 compared to stiffness group

*SVA* C7-sagittal vertical axis, *TSPM* total spinopelvic motion, *LL* lumbar lordosis, *PFA* proximal femur angle, *PT* pelvic tilt

### Effect of spinopelvic stiffness

In the standing position, the stiffness group had pelvises with more retroversion (higher PT; *p* < 0.001) and hips with more extension (higher PFA; *p* = 0.007) than the normal group (Table 1). Under the compensation of the pelvis and hip joints, the two groups showed a similar global balance.

When changing from the standing to the upright sitting position (Table 4), the stiffness group had 15° less pelvic retroversion as compared to the normal group (∆PT; *p* < 0.001) because of poor lumbosacral spine flexibility (∆LL: − 7° and − 25°, respectively; *p* < 0.001). To reach the upright sitting position, the stiffness group needed 15° more hip motion (∆PFA; *p* < 0.001). Trunk global balance showed an anterior shift to SVA approximately 6 cm while sitting in both groups (∆SVA: 2.2 cm and 1.3 cm, respectively; *p* = 0.399). TSPM was also similar in the mean value of both groups.

### Paradoxical spinopelvic motion

**Fig. 6:**
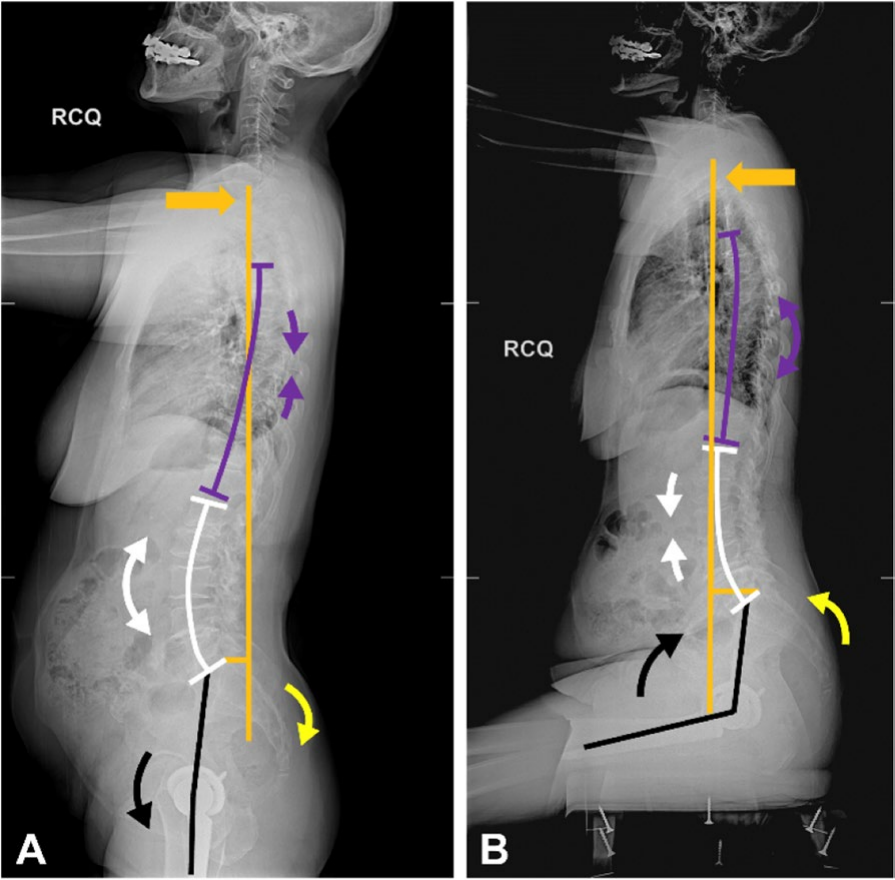
Paradoxical patient # 1. This was a 70 year old female. Standing SVA was negative (− 1.9 cm), and the trunk anterior shift from standing (**A**) to sitting (**B**) was 5.8 cm (orange arrow). The lumbar spine degeneration resulted in only 6° of lumbosacral motion (white arrows). The hip motion was − 77.2° (black arrow), which caused pelvic anterior tilt for 4.6° (yellow arrow). The anteriorly tilted pelvis led to reduced acetabular anterior clearance when sitting down. Moreover, as the THA cup version was not large enough (anteversion: 21°; inclination: 25°), posterior dislocation occurred 1 month after surgery. SVA, C7-sagittal vertical axis

**Fig. 7:**
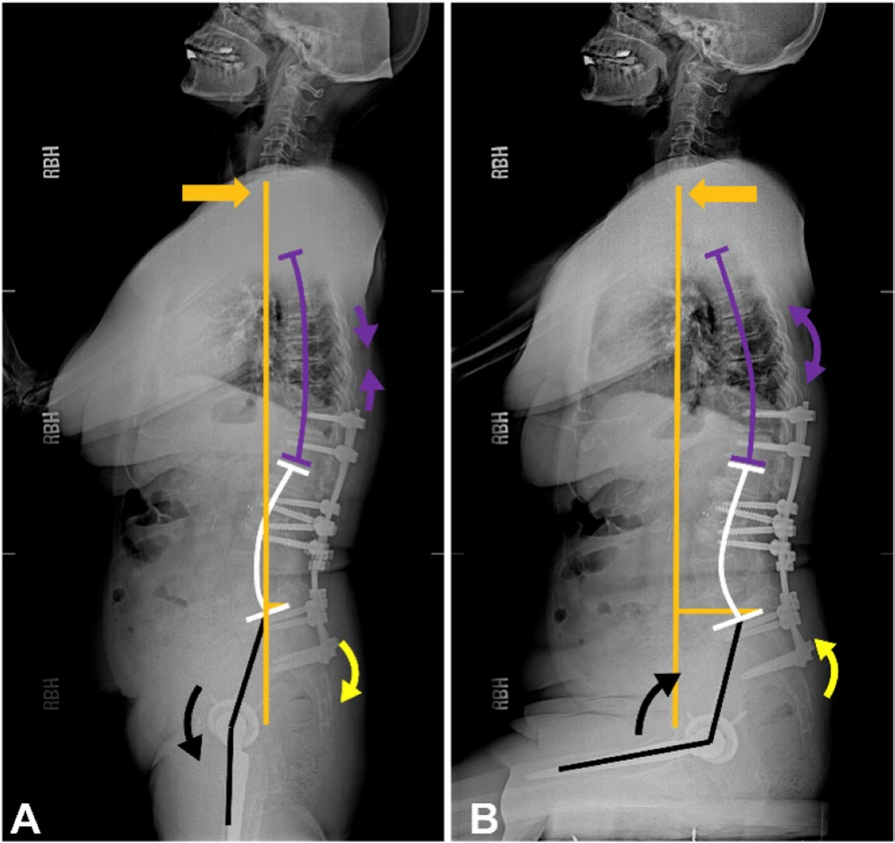
Paradoxical patient #5. This was a 76-year-old female with a balanced spine with SVA 2.2 cm. The trunk anterior shift from standing (**A**) to sitting (**B**) was 4.9 cm (orange arrow). An extensive spinal fusion of the lumbar spine (T12-S2) caused a fixed lumbosacral spine (∆LL: 0°). The hip motion was − 78.6° (black arrows), which caused pelvic anterior tilt for 2.4° (yellow arrow). The anterior tilt of the pelvis caused a decrease in acetabular anterior clearance in the sitting position. Moreover, as the THA cup version was not large enough (anteversion: 21°; inclination: 45°), posterior dislocation occurred 4 months after surgery. SVA, C7-sagittal vertical axis; LL, lumbar lordosis

Five patients had opposite pelvic and hip motions during the position change (Tables 1, 2, and 4, and Figs. 6 and 7). Such conditions were paradoxical spinopelvic motion. Among the five patients, two developed posterior dislocation after THA (1 and 4 months after THA, respectively, and 1 had previous spinal fusion before THA). Although the lumbosacral motion (∆LL) was similar to the stiffness group (*p* = 1.00), ∆PT was − 3° in patients with paradoxical motion (*p* = 0.015, as compared to the stiffness group) (Table 4).

In the standing position, the paradoxical group illustrated a balanced global trunk alignment (SVA: − 1.0 ± 5.1 cm) (Table 1). However, they had a similar sitting SVA to the stiffness group; thus, they had nearly 5 cm more of antecedent trunk shift as compared to the stiffness group when switching from the standing to sitting position (∆SVA: 1.3 cm and 6.2 cm, respectively, *p* = 0.014). Therefore, due to stiffness of the lumbosacral spine, the highest hip motion (∆PFA: − 78°; *p* < 0.017 as compared to the stiffness group) was compensated for this high degree of trunk shifting.

## Discussion

Spinopelvic stiffness is a well-established spinopelvic factor causing THA dislocation [4–8]. In this study, all patients with dislocation were in either the stiffness or paradoxical groups. In the case of reduced lumbosacral mobility resulting from a biological (e.g., degenerative disease) [9, 20] or an iatrogenic fusion [21–25] causing the recruitment of more hip motion for posture changes, and while reduced lumbosacral motion prevents the pelvis from providing corresponding anterior or posterior clearance for the femur, it may result in THA impingement and instability. According to Stefl et al.’s classification [7] and related studies [4, 6, 8], spinopelvic stiffness can be divided into three clinical conditions based on the orientation of the fixed pelvis to provide good surgical guidance. However, the influence of global alignment on spinopelvic motion was not investigated in their studies. Here, we analyzed the effect of global alignment on spinopelvic motions in a cohort study constituting 124 patients who underwent THA.

The major finding of this study was that the lumbosacral and hip motions were the major contributors to trunk shifting from a standing to sitting position. To the best of our knowledge, this is the first study to establish the influence of global balance on the spinopelvic motion.

Although ΔSVA was not directly correlated to ΔPT, it would have a direct effect on ΔLL and ΔPFA, and both parameters correlate to ΔPT (Fig. 5A and B). According to the multivariable regression analysis and regression model (ΔSVA = − 11.97 + 0.05ΔTK – 0.23ΔLL - 0.17ΔPFA) (Table 3), lumbosacral (*p* < 0.001) and hip motion (*p* < 0.001) are the main contributors to trunk motion. The lumbosacral motion has a greater impact on trunk shifting than hip motion. From this equation, to achieve the same extent of trunk shifting, a reduction in lumbosacral motion by 1° has to be compensated by an increase in hip motion by approximately 1.4°. Previous studies showed a negative linear correlation between hip and spine motions [9, 20]. However, the extent of trunk shifting required when changing postures varies on an individual basis, and it may also affect spinopelvic motion.

The influence of global trunk alignment was likely to be less significant among those with normal spinopelvic motion and those who can recruit greater lumbosacral motion and avoid excessive hip motion. It was particularly worth noting that some of the subjects with normal spinopelvic motion were clustered in the low ΔLL and low ΔSVA region since lower ΔLL was required for their lower ΔSVA (Fig. 3B). As a result, no excessive hip motion was recruited, thus maintaining ΔPT.

In contrast, global trunk alignment may have a greater impact on patients with spinopelvic stiffness. According to a recent study [19], spinopelvic stiffness was more prevalent in patients with kyphotic and imbalanced sagittal alignment with higher SVA. Due to higher standing SVA, patients with a kyphotic and imbalanced spine usually experience a smaller extent of trunk shifting from standing to sitting positions. This adhered to the findings of a recent work by Buckland et al. (ΔSVA = 4.9, 3.0, and 3.2 cm in normal, degenerative, and flatback spine respectively, *P* < 0.01) [26], and with the present study (Table 4). However, depending on the type of sagittal alignment [15], different spinal degenerative cascades would occur [27]. The pelvis was sometimes fixed in the anteverted position and exhibited hyperlordotic lumbar spine (i.e., “stuck standing” [6]). These patients tended to have balanced sagittal parameters and SVA when standing. Therefore, if a greater extent of SVA change was imperative from standing to sitting positions, due to stiffness of the lumbosacral spine, a greater hip motion might be recruited in these patients than those with a kyphotic and imbalanced stiff spine. According to the regression model, patients with a stiff spine (Tables 1 and 4, mean ΔTK = 1° and mean ΔLL = -7°), whose anterior trunk shift (ΔSVA) was up to 2.1 cm, might recruit a hip flexion of up to 72.7° from standing to sitting positions. This was consistent with the cut-off value corresponding to 0° pelvic tilt posture change.

We found that paradoxical spinopelvic motion was a very rare clinical variant of spinopelvic stiffness requiring special attention (Tables 1, 2, and 4) (Figs. 6 and 7). Although they only accounted for 4% of all THA cases, the dislocation rate was relatively high. To the best of our knowledge, no study has identified this phenomenon, except in a previous study [9] reporting that 3% of these outliers showed anterior pelvic tilt while sitting; however, potential mechanics were not investigated. Opposite motions of the hip joint and pelvis reduced acetabular anterior clearance in the sitting position, and also reduced the posterior clearance in the standing position. This placed patients constantly at high risk of mechanical impingement.

Global trunk alignment may be associated with paradoxical motions. Although their lumbosacral flexibility (ΔLL) was similar to that of the stiffness group (Table 4), balanced global alignment or negative SVA were observed during the standing position. When sitting, the C7 plumb line would move forward to about SVA 5 to 6 cm in both groups to achieve a comfortable upright sitting position, causing a relatively great amount of ΔSVA in patients in the paradoxical group. Figure 3B shows that the subjects with paradoxical motions are mostly in the low ΔLL and high ΔSVA region, which implies a disproportionally high extent of trunk shifting in the stiff lumbosacral spine. However, patients in the stiffness group demonstrated no paradoxical motion because the ΔSVA was not excessively high.

Therefore, we speculated that paradoxical motion might be related to a disproportionately high extent of trunk shift in the stiff lumbosacral spine, while the stiff lumbosacral motion segments could not provide sufficient ΔSVA. Therefore, excessive motion of the hip joint (mean ∆PFA = − 78°, *p* < 0.001 as compared to normal and stiffness group) might be their compensation mechanism. The conditions of these five patients (Table 2) could be classified into the following three clinical conditions:

1. Negative standing SVA with stiff lumbosacral spine (patients #1, #2, and #3): As shown in Fig. 6, patient #1 had lumbosacral stiffness because of severe degeneration in L2–3, L4–5, and L5-S1 segments. The standing SVA was − 1.9 cm. From standing to sitting positions, the C7 plumb line moved forward by 5.8 cm while the lumbosacral motion segments only provided − 6° motion. This forced the hip joint to increase its motion (∆PFA = − 77°) and provide the required extent of the trunk anterior shift to achieve trunk balance in the upright sitting position. This excessive hip motion caused a − 5° PT change during a position change.
2. Obesity with high sitting SVA (patient #4): Although the standing SVA of this patient was 5.4 cm, the sitting SVA (9.4 cm) was relatively high due to obesity (BMI: 35 kg/m^2^). From standing to sitting positions, the C7 plumb line moved 4 cm forward. However, because of the stiffness caused by L4–5 spinal fusion, the lumbosacral motion segments only provided − 4° motion. This forced the hip joint to increase its motion (∆PFA = − 86°) to allow the pelvis to tilt forward with the spine and provide the required trunk anterior shift. This eventually resulted in a PT change of − 3° during the position change.
3. Balanced global alignment with a fixed lumbosacral spine (patient #5): As shown in Fig. 7, due to the extensive thoraco-lumbo-sacral fusion, this patient had a fixed lumbosacral spine (ΔLL = 0°), and could only increase the hip joint motion to provide 4.9 cm of trunk anterior shift. This resulted in a PT change of − 2° in the sitting position.

Due to the greatest hip joint motion recruitment, opposite hip joint and pelvic motions when changing postures in patients with paradoxical motion if the component version is not properly placed, it would have a higher chance of THA instability than spinopelvic stiffness; otherwise, a dual-mobility cup might be considered.

This study had some limitations. First, its retrospective nature limited both the surgical approach’s consistency and the cup version of five surgeons, potentially affecting THA stability [4]. Therefore, we focused on the analysis of spinopelvic motions and global trunk alignment, rather than discussing dislocation cause. Second, because of the low prevalence of paradoxical motion, only five paradoxical patients out of 124 patients were identified. Further analysis, such as the risk factors that contributed to paradoxical motion could not be performed. More paradoxical patients need to be included for further analysis. Third, we were not able to identify a directed correlation between ΔSVA and ΔPT. Nevertheless, we constructed a multivariable model of ΔSVA to other sagittal parameters, including ΔLL and ΔPFA, which were the most important parameters that contribute to ΔPT. This might be reasonable because of a negative correlation between ΔLL and ΔPT, and a positive correlation between ΔPFA and ΔPT (Fig. 5A and B). However, ΔLL and ΔPFA have a synergic effect on ΔSVA. Fourth, we used digital triple-film radiographs instead of EOS images. Theoretically, digital radiography can distort the image-boundary and increase the angle measurement error; however, no study has confirmed a significant difference between the two approaches. Finally, recent studies found that a combined sagittal index (sum of the cup ante-inclination and PFA) can effectively predict THA dislocation [4, 6, 8]; however, this study did not include this index in the measurement. Therefore, larger scale studies are suggested.

## Conclusion

Global trunk alignment and spinopelvic motion displayed a statistically significant linear correlation. The major contributors to global alignment postural change were lumbosacral and hip motion. Paradoxical spinopelvic motion is a rare but dangerous clinical condition in THA. This phenomenon may be related to a disproportional high extent shifting of the trunk gravity line in patients with stiff lumbosacral spine, recruiting excessive hip joint motion while changing positions than other stiffness subjects. The opposite directions of the hip joint and pelvic movements during position change would decrease acetabular clearance for the proximal femur, which would have a higher risk of THA instability. Further larger-scale studies are needed to identify the risk factors contributing to paradoxical motion.

## Data Availability

The datasets used and/or analyzed during the current study are available from the corresponding author on reasonable request.

## Abbreviations

THA: Total hip arthroplasty
PT: Pelvic tilt
SS: Sacral slope
PI: Pelvic incidence
TK: Thoracic kyphosis
LL: Lumbar lordosis
SVA: Sagittal vertical axis
PFA: Pelvic-femoral angle
TSPM: Total spinopelvic motion
